# Sex difference in the progression of manic symptoms during acute hospitalization: A prospective pilot study

**DOI:** 10.1002/brb3.1568

**Published:** 2020-02-13

**Authors:** Osama A. Abulseoud, Güliz Şenormancı, Ömer Şenormancı, Oya Güçlü, Brooke Schleyer, Ulas Camsari

**Affiliations:** ^1^ Neuroimaging Research Branch IRP National Institute on Drug Abuse Baltimore MD USA; ^2^ University of Health Sciences Bursa Yüksek İhtisas Training and Research Hospital Psychiatry Department Bursa Turkey; ^3^ Bakirkoy Research & Training Hospital for Psychiatry, Neurology, Neurosurgery and Psychiatry Department İstanbul Turkey; ^4^ Department of Psychiatry and Psychology Mayo Clinic Rochester MN USA

**Keywords:** aggressive and disruptive behavior, insight, mania, phenotype, sex difference, sexual interest, Young Mania Rating Scale

## Abstract

**Objectives:**

Acute mania is a serious medical condition that impacts men and women equally. Longtime presentation of manic symptoms is sex‐dependent; however, little is known about acute symptoms of mania. The objective of this study is to track and compare acute manic symptoms for sex differences during inpatient hospitalization.

**Methods:**

All patients with bipolar mania admitted to a large university hospital between January and October 2017 were invited to participate in this longitudinal naturalistic follow‐up study. Manic (YMRS), depressive (MADRS), and psychotic (PAS) symptoms were tracked daily from admission to discharge.

**Results:**

The total YMRS scores decreased significantly overtime (*p* < .0001) in both male (*n* = 34) and female (*n* = 23) patients (*p* = .7). However, male patients scored significantly higher in sexual interest (*p* = .01), disruptive and aggressive behavior (*p* = .01), and appearance (*p* < .001) while females had better insight into their illness (*p* = .01). Males and females received similar doses of lithium (*p* = .1), but males received significantly higher doses of valproic acid (VPA) in comparison with females (*p* = .003). However, plasma lithium and VPA concentrations at discharge were not significantly different between sexes.

**Conclusion:**

Our results show sex differences in the progression of certain domains of manic symptoms in a cohort of 23 female and 34 male patients admitted to a large academic center in Turkey. Males, in this sample, exhibited more sexual interest, disruptive and aggressive behaviors, better grooming, and less insight compared to females. While these results are concordant with our preclinical findings and with anecdotal clinical observations, replication in larger samples is needed.

## Graphical abstract

The progression of certain domains of manic symptoms shows sex difference. Female manic patients had better insight into their illness compared to manic males who exhibited more sexual interest, disruptive, and aggressive behaviors, but better appearance. Both males and females received comparable doses of antipsychotics and lithium. Valproic acid (VPA) doses were significantly higher in males compared to females. However, VPA plasma concentrations were not significantly different between the two groups.

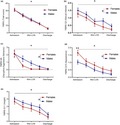

## SIGNIFICANT OUTCOMES

1. The progression of certain domains of manic symptoms shows sex difference.
2. Female manic patients had better insight into their illness compared to manic males who exhibited more sexual interest, disruptive, and aggressive behaviors, but better appearance.
3. Both males and females received comparable doses of antipsychotics and lithium. Valproic acid (VPA) doses were significantly higher in males compared to females. However, VPA plasma concentrations were not significantly different between the two groups.

## LIMITATIONS

1. Despite successfully enrolling 57 acutely manic patients in the study, the sample size is still small.
2. Establishing the study in a non‐HMO hospital in Turkey allowed for longer hospital stays. However, as a nonwestern country, the prevalence of comorbid alcohol and substance use disorders was low.
3. Diagnosis was achieved through clinical interviews, not structured interviews.

## 1 INTRODUCTION

Bipolar disorder type I (BPI) is a serious medical condition characterized by the occurrence of one or more manic episodes. Lifetime prevalence of BPI is roughly the same in both males and females (Grant et al., 2005; Merikangas et al., 2007; Mitchell, Slade, & Andrews, 2004; Weissman et al., 1988). However, despite equal sex ratio in prevalence, men have significantly earlier age of onset of first mania (Grant et al., 2005; Kennedy et al., 2005), more substance use (Kessing, 2004; Nivoli et al., 2011), behavioral (Kawa et al., 2005), and legal problems (Baldassano et al., 2005), and are more likely to be treated with lithium (Karanti et al., 2015). BPI women, on the other hand, have shorter interval to the evolution of manic episodes (Kumar & Ram, 2001) and less insight into their illness (Guclu, Karaca, Yildirim, & Ozkose, 2011), but more rapid cycling (Slyepchenko et al., 2017; Tondo & Baldessarini, 1998), mixed mania (Arnold, McElroy, & Keck, 2000; Diflorio & Jones, 2010; Sato, Bottlender, Kleindienst, & Möller, 2002), longer (Hendrick, Altshuler, Gitlin, Delrahim, & Hammen, 2000) and more frequent hospitalizations for mania (Winokur et al., 1994), and slightly better response to lithium treatment (Viguera, Tondo, & Baldessarini, 2000). Additionally, BPI women are more likely to have lifetime history of attempted (Nivoli et al., 2011) and completed suicide (Clements et al., 2013). In contrast to these lifetime differences between male and female bipolar patients, sex differences in acute manic manifestations have received little attention. In a cross‐sectional study, Young, Biggs, Ziegler, and Meyer (1978) assessed acute mania severity upon psychiatric admission using Young Mania Rating Scale (YMRS) in 149 consecutive patients and found that males scored significantly higher on the sexual interest item than female patients (Young et al., 2007). This study suggests subtle sex differences in the symptomatology of acute mania. However, only YMRS scores at admission were compared between subjects without further exploration of sex differences amid the course of manic symptoms during hospitalization or whether males and females received similar treatment.

Sex differences, in general, stem from a complex interplay between neurobiological and psychosocial factors. To explore the neurobiological aspect, we used our newly established lateral hypothalamic‐kindled (LHK) rat mania model to examine sex differences in manic‐like behaviors during mania induction (Abulseoud et al., 2014) and we found that male rats exhibited more sexual behavior, significantly increased locomotor activity during the light phase, and reduced rest interval, while females displayed significantly higher ethanol consumption and more frequent rearing behaviors (Abulseoud et al., 2015). To test whether these results translate in a clinical model, we sought to examine sex difference in the progression of manic symptoms during acute hospitalization in an academic medical center in Turkey. We chose such a facility to collaborate with because of their expertise in diagnosis, symptom quantification and treatment of acute mania, and the large volume of patients, and equally important, because of the unrestricted hospital length of stay. We hypothesized, based upon past research on acute mania (Hendrick et al., 2000; Kumar & Ram, 2001; Viguera et al., 2000; Young et al., 2007) and our work (Abulseoud et al., 2015), that the presentation of acute manic symptoms is sex‐dependent. Specifically, males will exhibit more aggressive and sexual manifestations compared to females. Our results document sex differences in several individual manic manifestations.

## 2 METHODS

Study protocol was approved by the institutional review boards of the University of Health Sciences Bursa Yuksek Ihtisas Training and Research Hospital and Bakirkoy Research and Training Hospital for Psychiatry, Neurology and Neurosurgery, İstanbul, Turkey. The University hospital is a large district hospital that serves the southern Marmara region with a population of about 4 million. The adult inpatient psychiatric unit has 110 beds (37 for females). All patients admitted to the unit between January and October 2017 and screened for history of bipolar diagnosis and current manic episode symptoms were invited to participate in this prospective naturalistic study. We excluded patients with schizoaffective disorder, intellectual disability, or illiteracy. Pregnant or lactating women were also excluded from the study. Study procedures were explained in detail for potential participants. Those who agreed to participate signed an informed consent and underwent detailed clinical interview by two Turkish board certified psychiatrists (GS and OS) assisted with two senior psychiatry residents. All cases were discussed prior to enrollment to confirm bipolar manic diagnosis. Physical examination, baseline laboratories including serum electrolytes, complete blood count, lipid panel, thyroid, liver, and kidney function tests were performed in all patients as part of routine work‐up. Plasma valproate (VPA) levels were measured by color spectrophotometry (Belal, El‐Kafrawy, Mahrous, Abdel‐Khalek, & Abo‐Gharam, 2016), and lithium levels are measured by flame photometric method (Levy & Katz, 1970). Blood samples were collected 12 hr after the last dose.

Manic, depressive, and psychotic symptoms were tracked daily throughout the hospital length of stay (LOS) by the same team. To ensure consistency between different raters, the study team members had daily discussions about the ratings for individual study participants. We used Young Mania Rating Scale (YMRS; Young et al., 1978), Montgomery–Aberg Depression Rating Scale (MADRS; Montgomery & Asberg, 1979), and the Scale for the Assessment of Positive Symptoms (SAPS; Andreasen, 1984). YMRS is an 11‐item clinician rating scale, each item is given a severity rating. There are four items that are graded on a 0 to 8 scale (irritability, speech, thought content, and disruptive/aggressive behavior), while the remaining seven items are graded on a 0 to 4 scale. MADRS is a 10‐item scale: 1. Apparent sadness 2. Reported sadness 3. Inner tension 4. Reduced sleep 5. Reduced appetite 6. Concentration difficulties 7. Lassitude 8. Inability to feel 9. Pessimistic thoughts 10. Suicidal thoughts. Each item is scored 0–6. SAPS consists of 34 items divided into four positive symptom subscales: hallucinations, delusions, bizarre behavior, and positive formal thought disorder. Each subscale includes a global rating scale. In addition, detailed records for daily treatment and vital signs were also collected. Medication doses were recorded on daily basis throughout the hospital LOS.

### 2.1 Statistical analysis

All data were presented as means (±*SEM*). Data were examined for normal distribution. Demographic and baseline laboratory data were examined for sex difference using student *t* test or Fisher exact test/ chi‐square statistics as appropriate. Medications were grouped into lithium, valproic acid (VPA), antipsychotic, benzodiazepine, and other categories upon admission and at time of discharge. Antipsychotic medications were converted into chlorpromazine equivalent dose according to the classical mean dose (Leucht et al., 2015) and the minimum effective dose methods (Leucht et al., 2014). Benzodiazepines were converted into lorazepam dose equivalent according to published tables (Brett & Murnion, 2015). Separate two‐factor ANOVAs were used to examine sex and hospitalization effects on different medication classes. Rating scale scores were adjusted to hospital LOS into five time points: admission, 25% LOS, mid‐LOS, 75% LOS, and discharge scores. Mean total scores (YMRS, MADRS, and SAPS) and individual items (YMRS and MADRS) were compared between male and female patients using separate repeated measures ANOVAs. Analysis was done using GraphPad Prism V7 software. Results are considered significant when *p* < .05.

## 3 RESULTS

### 3.1 Demographic information

A total of 72 patients (females: *n* = 30) with bipolar manic episode were admitted to the inpatient unit during the study interval from January to October 2017. A total of 58 patients (females: *n* = 24) consented for the study. Patients reported mild (MADRS < 19) to moderate (MADRS < 30) depressive symptoms. However, none met DSM‐5 criteria for mixed episode as defined by having at least three out of six concurrent depressive symptoms during the majority of manic episode days (American Psychiatric Association, 2013). The patients who refused to participate in the study (total *n* = 14 [males: *n* = 8]) had difficulty understanding the study process and provide informed consent due to severe agitation or delirious mania. One female patient was discharged to another facility after 3 days and was excluded from analysis. The final cohort consisted of 34 males and 23 females. As shown in Table 1, the mean (±*SEM*) age was 37.6 (2.3) and 40.9 (2.4) years for males and females, respectively. Most of the females were married (65%) and homemakers (57%), while about half of male patients were married (50%), employed, or students (53%). More males compared to females reported legal problems (M vs. F: 40.6% vs. 17.4%, *p* = ns) and a past history of alcohol use (M vs. F: 51% vs. 13%, *p* = .004). The majority of males (68%) and females (83%) were classified as medium socioeconomic status based on reported monthly income (Low socioeconomic level = up to 999 Turkish Lera [TL], medium = 1,000–1,999 TL, and high = 2,000 TL or over).

**Table 1:** Demographic information

	Males (*n* = 34)	Females (*n* = 23)	*p* value
Age (years)	37.6 ± 2.3	40.9 ± 2.4	.37
Education (years)	10.42 ± 0.7	9.2 ± 1.0	.24
Marital status (married)	17 (50%)	15 (65%)	.28
Medium socioeconomic status	23 (68%)	19 (83%)	.23
Employment (full time employed or student)	18 (53%)	4 (17%)	.07
Homemaker		13 (57%)	
Legal issues	13 (40.6%)	4 (17.4%)	.14
History of alcohol use	16 (51.6%)	3 (13%)	.004

### 3.2 Bipolar disorder history

As detailed in Table 2, the hospital LOS did not vary significantly between males (20.7 ± 2.1) and females (22.6 ± 2.3) and about 2/3 of patients presented with psychotic features. Similarly, the age of first hospitalization did not differ between males and females. However, females had a nonsignificant trend (*p* = .08) toward more previous hospitalizations, but no differences in the number of prior manic or depressive episodes or the presence of psychotic features during past manic episodes or in the history of aggressive behaviors. Females, however, reported significantly more previous suicidal attempts compared to males (*p* = .002).

**Table 2:** Bipolar disorder history

	Males (*n* = 34)	Females (*n* = 23)	*p* value
Hospital LOS (days)	20.7 ± 2.1	22.6 ± 2.3	.53
Psychotic features during current manic episode	20 (64.5%)	14 (60.9%)	.99
Age of first hospitalization	29.6 ± 2.03	29.5 ± 2.6	.99
Number of prior hospitalizations	3.78 ± 0.54	5.5 ± 0.87	.08
Number of prior severe manic episodes	4.12 ± 0.6	5.8 ± 0.9	.11
Number of prior severe depressive episodes	2.77 ± 0.44	4.45 ± 0.97	.08
Psychotic features during past manic episodes	25 (83.3%)	18 (78.3%)	.76
History of verbal aggressive behavior	13 (41.9%)	8 (34.8%)	.99
History of physical aggressive behavior	16 (51.6%)	13 (56.5%)	.59
History of previous suicide attempts	3 (9.4%)	11 (47.8%)	.002

### 3.3 Baseline medical history, vital signs, and laboratory results

Other than bipolar illness, the cohort was generally healthy, and only few patients had comorbid medical conditions: diabetes (*n* = 1), hypertension (*n* = 1), and morbid obesity (*n* = 1). Males had slight but significant increase in heart rate and diastolic blood pressure and elevated, but within normal range, liver functions (ALT, AST, and direct bilirubin), alongside reduced vitamin B_12_ concentration. On the other hand, females had mild anemia (low Hgb, HCT, and RBC counts) and lower free T3 concentration. No significant sex differences were observed in other vital signs or laboratory measures (Table 3).

**Table 3:** Baseline vital signs and laboratory results

Variable and normal range	Males (*n* = 34)	Females (*n* = 23)
Vital signs		
Heart rate* (60–100 beat/min)	85.3 ± 1.8	79.7 ± 1.6
Systolic BP (90–120 mmHg)	118.2 ± 1.8	115.7 ± 2.1
Diastolic BP* (60–80 mmHg)	74.9 ± 1.1	70.9 ± 1.5
Temperature (36.1−37.2°C)	36.5 ± 0.06	36.5 ± 0.04
Serum electrolytes		
Sodium (136–145 mEq/L)	138.9 ± 0.57	138 ± 0.4447
Potassium (3.5–5.0 mEq/L)	4.26 ± 0.08	4.18 ± 0.11
Chloride (98–106 mEq/L)	104.3 ± 0.58	105.2 ± 0.73
Magnesium (1.5–2.4 mg/dl)	2.108 ± 0.04	2.04 ± 0.04
Fasting blood glucose		
Glucose (70–100 mg/dl)	107.3 ± 8.18	97.19 ± 3.26
Complete blood count		
White blood cells (WBC: ×10^9^/L)	8.71 ± 0.46	9.16 ± 0.47
Red blood cells*** (RBC: 4.0–10.0 × 10^6^/µl)	4.995 ± 0.1	4.34 ± 0.08
Hemoglobin*** (14–17 g/dl)	14.37 ± 0.22	12.23 ± 0.29
Hematocrit*** (41−51%)	43.49 ± 0.78	36.77 ± 0.84
Platelet count (150−350 × 10^9^/L)	253.3 ± 11.33	274.7 ± 21.25
Mean corpuscular volume (MCV: 80−100 fl)	87.27 ± 0.74	84.6 ± 0.95
Mean corpuscular hemoglobin (MCH: 28−32 pg)	28.89 ± 0.28	28.16 ± 0.4
Mean corpuscular hemoglobin concentration MCHC (32−36 g/dl)	33.11 ± 0.13	33.26 ± 0.15
Red cell distribution width (RDW 11.8−14.5%)	12.82 ± 0.12	13.57 ± 0.32
Lipid profile		
Total cholesterol (150–199 mg/dl)	161.9 ± 8.36	153.8 ± 6.34
High‐density lipoprotein HDL (40–59 mg/dl)	45.22 ± 2.22	48.06 ± 2.65
Low‐density lipoprotein LDL (≤130 mg/dl)	90.4 ± 5.91	80.22 ± 5.03
Triglyceride (≤150 mg/dl)	131.5 ± 16.7	127.7 ± 17.26
Very low‐density lipoprotein (2−30 mg/dl)	26.29 ± 3.34	25.54 ± 3.44
Liver function		
Aspartate aminotransferase* (AST: 0−35 U/L)	38.16 ± 4.42	23.35 ± 3.68
Alanine aminotransferase** (ALT: 0−35 U/L)	38.83 ± 5.37	18.94 ± 1.83
Alkaline phosphatase (36–92 U/L)	61.64 ± 4.04	56.67 ± 4.65
Albumin (3.5–5.5 g/dl)	4.04 ± 0.15	3.783 ± 0.06
Total bilirubin (0.3–1.2 mg/dl)	0.72 ± 0.07	0.54 ± 0.05
Direct bilirubin* (0–0.3 mg/dl)	0.13 ± 0.00	0.1 ± 0.01
Indirect bilirubin (0.3–0.9 mg/dl)	0.53 ± 0.08	0.72 ± 0.21
Vit B_12_ ** (200−800 pg/ml)	244.3 ± 15.32	420.9 ± 57.51
Serum amylase (U/L)	46 ± 5.36	39.38 ± 3.43
Kidney function		
Blood urea nitrogen (BUN: 8–20 mg/dl)	11.75 ± 0.72	12.17 ± 1.74
Serum creatinine (0.7–1.3 mg/dl)	0.91 ± 0.04	0.78 ± 0.1
Thyroid function		
Thyroid stimulating hormone (0.5–5.0 TSH: µU/ml)	1.73 ± 0.28	1.82 ± 0.26
Free T4 (0.9–2.4 ng/dl)	1.16 ± 0.04	1.27 ± 0.06
Free T3** (3.6–5.6 ng/L)	3.32 ± 0.08	2.93 ± 0.12

* *p* < .05,

** *p* < .01,

*** *p* < .001.

### 3.4 Medications upon admission and at time of discharge

Table 4 describes the details of medications used. All patients were either on antipsychotic medication upon admission or were started on one during hospitalization. Quetiapine and haloperidol were the most commonly prescribed antipsychotics. We used classical mean dose (Leucht et al., 2015) and the minimum effective dose methods (Leucht et al., 2014) to convert individual antipsychotic daily doses to chlorpromazine equivalent dose. The mean dose equivalent did not differ between males and females upon admission or at discharge. Antipsychotic dose equivalent also did not show significant change during hospitalization [*F* (1, 107) = 0.1057, *p* = .7] in both sexes [*F* (1, 107) = 2.722, *p* = .1], and no interaction between hospitalization and sex was observed [*F* (1, 107) = 0.452, *p* = .5].

**Table 4:** Medications: Number and percentage of patients receiving antipsychotic (chlorpromazine dose equivalent), lithium dose and plasma concentration, valproic acid (VPA) dose and plasma concentration, and benzodiazepine dose upon admission and at time of discharge. Significant sex and hospitalization effects for VPA by Two‐factor ANOVA

Antipsychotic (Chlorpromazine equivalent)				
	Admission		Discharge	
	*N* (%)	Daily dose (mg)	*N* (%)	Daily dose (mg)
Males (*n* = 34)	33 (97%)	898.9 ± 130.2	34 (100%)	931.7 ± 75.7
Females (*n* = 23)	22 (100%)	806.3 ± 86.1	22 (100%)	711.9 ± 85.6

Lithium					
	Admission		Discharge		
	*N* (%)	Daily dose (mg)	*N* (%)	Daily dose (mg)	Plasma Conc.
Males (*n* = 34)	9 (26%)	933.3 ± 92.8	18 (53%)	1,103 ± 64.5	0.79 ± 0.06
Females (*n* = 23)	9 (41%)	844.4 ± 98.7	13 (59%)	830.8 ± 102.8	0.66 ± 0.06

Valproic acid (VPA)					
	Admission		Discharge		
	*N* (%)	Daily dose (mg)	*N* (%)	Daily dose (mg)	Plasma Conc.
Males (*n* = 34)	10 (29%)	975 ± 25	17 (50%)	1,309 ± 94.7	76 ± 5.2
Females (*n* = 23)	8 (37%)	737.5 ± 90.5	11 (50%)	977.3 ± 85.6	73.1 ± 6.8

Benzodiazepine (Lorazepam equivalent)				
	Admission		Discharge	
	*N* (%)	Daily dose (mg)	*N* (%)	Daily dose (mg)
Males (*n* = 34)	17 (50%)	2.7 ± 0.4	10 (29%)	2.8 ± 0.3
Females (*n* = 23)	14 (64%)	2.2 ± 0.2	11 (50%)	2 ± 0.3

In addition to antipsychotics, most patients were treated with standard mood stabilizers: lithium and valproic acid (VPA). Less males (26%) compared to females (41%) were prescribed lithium upon admission. However, the number of male patients on lithium doubled during hospitalization. At discharge, 53% of males and 59% of females were on lithium. Daily lithium dose did not differ significantly between males and females upon admission or at discharge. Two‐factor ANOVA shows a nonsignificant trend toward sex effect in lithium dose [*F* (1, 45) = 3.895, *p* = .054], but there was no difference between males' and females' plasma lithium concentration at time of discharge (*t* = 1.527, *p* = .14) and no effect for hospitalization on lithium dose [*F* (1, 45) = 0.7256, *p* = .3] or interaction between sex and hospitalization [*F* (1, 45) = 1.003, *p* = .3] were observed.

On the other hand, VPA dose showed significant effect for sex [*F* (1, 42) = 9.645, *p* = .003] and hospitalization [*F* (1, 42) = 9.8, *p* = .003], but no interaction between the two factors [*F* (1, 42) = 0.2635, *p* = .6]. Multiple comparison test shows significant difference between males and females at discharge (mean difference = −331.6, 95% CI = −651.3 to −11.78, *p* = .038) and between males at admission and males at discharge (mean difference = −333.8, 95% CI = −663.2 to −4.492, *p* = .045). However, despite this marked sex difference in dose, VPA plasma concentrations at time of discharge were not significantly different between males and females (*t* = 0.3557, *p* = .7).

Benzodiazepines were also used widely. Four benzodiazepines were used: lorazepam, clonazepam, alprazolam, and diazepam. We calculated lorazepam dose equivalent according to published tables (Brett & Murnion, 2015). We observed significant overall effect of sex [*F* (1, 48) = 4.132, *p* = .047] on benzodiazepine dose equivalent, but no specific differences between males and females at admission or discharge were detected by multiple comparison test. Hospitalization did not seem to have a significant effect on benzodiazepine dose [*F* (1, 48) = 0.01125, *p* = .9] and no interaction between sex and hospitalization [*F* (1, 48) = 0.09405, *p* = .7].

Besides antipsychotics, lithium, VPA, and benzodiazepines, other medications were used such as anticholinergics (*n* = 27), antidepressants: Sertraline (*n* = 1), Lamotrigine and Venlafaxine (*n* = 1), Propranolol (*n* = 2), and vitamin B complex (*n* = 1).

### 3.5 Course of manic manifestations (YMRS)

**Figure 1:**
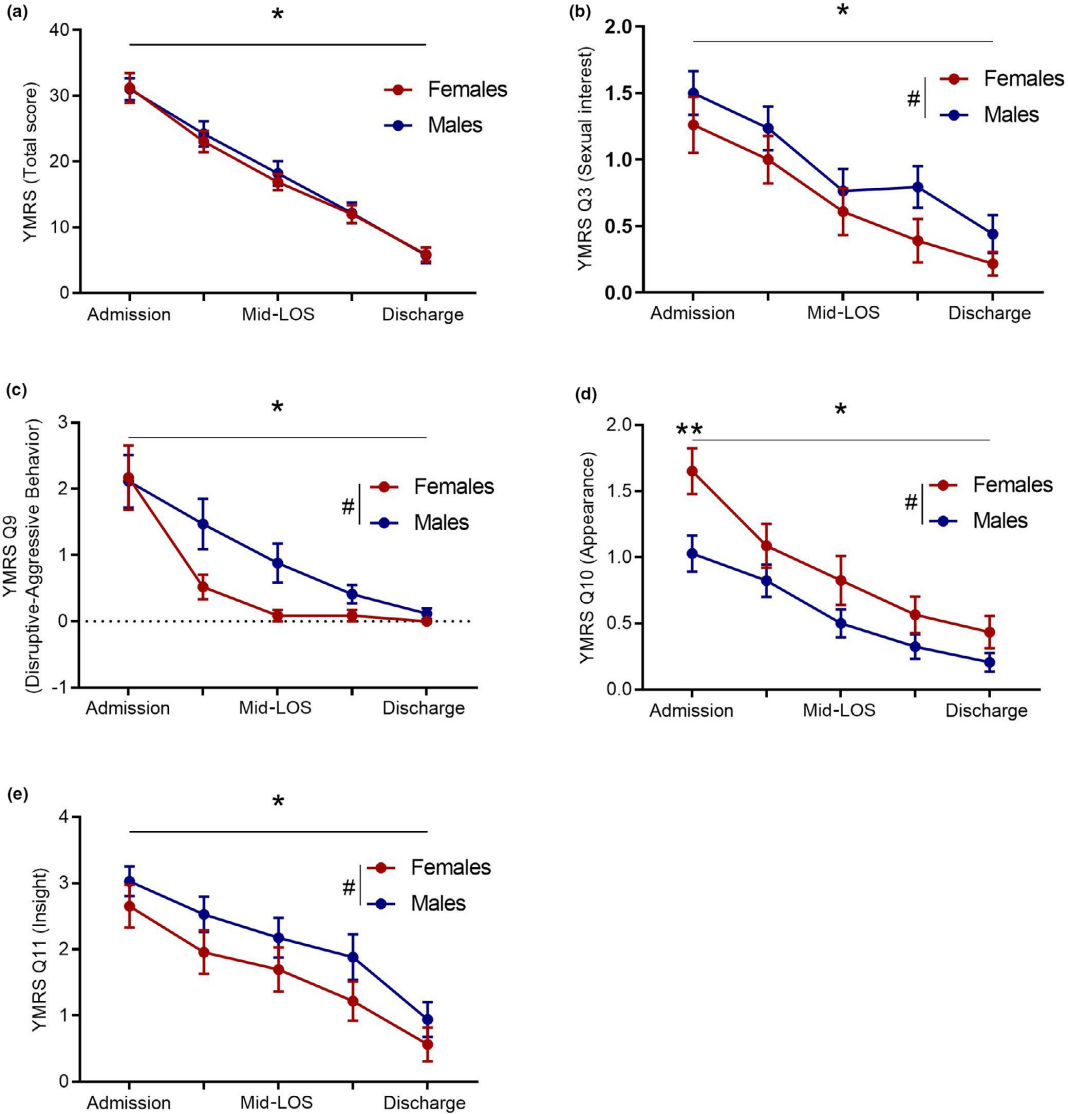
Progression of manic symptoms as measured by YMRS over hospital LOS in male and female patients by two‐way ANOVA. (a) Total YMRS score shows significant effect of time [*F* (4, 220) = 149.9, *p* < .0001], but not sex [*F* (1, 55) = 0.06176, *p* = .8] and no interaction between time and sex [*F* (4, 220) = 0.2012, *p* = .9. (b) YMRS Q3 sexual interest shows significant effect of time [*F* (4, 275) = 12.69, *p* < .0001], and sex [*F* (1, 275) = 5.625, *p* = .01] but no interaction between time and sex [*F* (4, 275) = 0.1477, *p* = .9]. The overall sex difference was not specific to a time point by Sidak's multiple comparison test. (c) YMRS Q9 disruptive and aggressive behavior shows significant effect of time [*F* (4, 275) = 17.32, *p* < .0001], and sex [*F* (1, 275) = 5.614, *p* = .018] but no interaction between time and sex [*F* (4, 275) = 1.156, *p* = .3]. The overall sex difference was not specific to a time point. However, a nonsignificant trend for sex difference was observed at the 25% LOS time point (mean difference = −0.09488, 95% CI of difference = −1.992 to 0.0941, *p* = .09) by Sidak's multiple comparison test. (d) YMRS Q10 appearance shows significant effect of time [*F* (4, 275) = 19.78, *p* < .0001], and sex [*F* (1, 275) = 16.56, *p* < .0001] but no interaction between time and sex [*F* (4, 275) = 0.789, *p* = .7]. In addition to the overall sex difference, significant (**) sex difference was observed at the admission time point [mean difference = 0.6228, 95% CI of difference = 0.143 to 1.103, *p* = .004) by Sidak's multiple comparison test. (e) YMRS Q11 insight also shows significant effect of time [*F* (4, 275) = 13.39, *p* < .0001], and sex [*F* (1, 275) = 6.717, *p* = .01] but no interaction between time and sex [*F* (4, 275) = 0.0868, *p* = .9]. (*) Indicates significant effect of time and (#) indicates significant effect of sex in all measures. The overall sex difference was not specific to a time point by Sidak's multiple comparison test

Daily YMRS ratings were collected and verified by two experienced psychiatrists (GS and OS) assisted with two senior residents. All rating results were discussed by the team to ensure reasonable agreement. The admission total YMRS scores ranged between 10 and 51 (mean ± *SEM*: 31.2 ± 2.2) for females and between 15 and 55 (mean ± *SEM*: 31.0 ± 1.7) for males suggesting moderate to severe mania. Since the hospital LOS varied widely between patients (5–60 days), we identified five time points: admission, 25% LOS, mid‐LOS, 75% LOS, and discharge. To examine the effect of sex and time on total and individual YMRS scores, we used repeated measures ANOVA. A significant effect of time [*F* (4, 220) = 149.9, *p* < .0001], but not sex [*F* (1, 55) = 0.06176, *p* = .8], and no interaction between time and sex [*F* (4, 220) = 0.2012, *p* = .9] were observed for the total YMRS scores (Figure 1a). However, sex differences were evident in four items: sexual interest [Q3; *F* (1, 275) = 5.625, *p* = .01, Figure 1b], disruptive and aggressive behavior [Q9; *F* (1, 275) = 5.614, *p* = .018, Figure 1c], appearance [Q10; *F* (1, 275) = 16.56, *p* < .001, Figure 1d], and insight [Q11; *F* (1, 275) = 6.717, *p* = .01, Figure 1e]. Specifically, males scored higher in sexual interest throughout hospital LOS, while both male and female patients scored higher in disruptive and aggressive behavior subscale upon admission and both showed complete resolution by end of hospitalization. However, female ratings dropped quickly as early as the first quarter while male ratings decreased steadily throughout the LOS. The opposite was observed for appearance, where lower scores are given for appropriate dress and grooming, and higher scores reflect disheveled and unkempt appearance. Unlike males, females had consistently poor appearance, specifically upon admission (*p* = .004), and it did not drop to zero at discharge. Despite poor appearance, females had better insight into their illness compared to males throughout the hospital LOS.

### 3.6 Course of depressive symptoms (MADRS)

Mild (MADRS < 19) to moderate (MADRS < 30) depressive symptoms were observed in both males (MADRS range: 4–25, mean ± *SEM* = 11.4 ± 0.8) and females (MADRS range: 0–26, mean ± *SEM* = 12.9 ± 1.3). Two‐factor ANOVA shows significant reduction in depressive symptoms over time [*F* (4, 270) = 30.36, *p* < .0001] with a nonsignificant trend toward lower scores in males throughout hospitalization [*F* (1, 270) = 2.778, *p* = .09]. There was no interaction between time and sex [*F* (4, 270) = 0.1735, *p* = .9]. No sex difference was observed in any of the individual MADRS items (data not shown).

### 3.7 Course of psychotic features (SAPS)

A wide range of psychotic symptom severity was observed upon admission in both males (SAPS range: 8–93, mean ± *SEM* = 25.3 ± 2.8) and females (SAPS range: 0–74, mean ± *SEM* = 0–74, 29.2 ± 3.2). Two‐factor ANOVA shows significant reduction in psychotic symptoms over time [*F* (4, 270) = 32.99, *p* < .0001] with no effect for sex [*F* (1, 270) = 0.080, *p* = .7] or interaction between time and sex [*F* (4, 270) = 0.4969, *p* = .7].

## 4 DISCUSSION

To the best of our knowledge, this is the first study that prospectively followed acute manic patients throughout hospitalization in a nonhealth maintenance organization (HMO). Our results show significant sex differences in four specific manic domains. Males scored higher in sexual interest, disruptive, and aggressive behaviors, while females had poor appearance but better insight into their illness. Both hypersexuality and aggressive behavior reflect some degree of impulsivity and poor cognitive control. Males in general tend to exhibit impulsive behaviors more than females (Cross, Copping, & Campbell, 2011) possibly due to variations in prefrontal cortical (PFC) function (Smaers, Mulvaney, Soligo, Zilles, & Amunts, 2012; Staiti et al., 2011) and connectivity (Chuang & Sun, 2014) between males and females. Past research has shown that bipolar manic patients also score very high in impulsivity scales (Strakowski et al., 2009; Swann, Pazzaglia, Nicholls, Dougherty, & Moeller, 2003). However, whether there are sex differences in impulsive behaviors among manic patients remain largely unknown. In agreement with our findings, one cross‐sectional study reported that male manic patients scored significantly higher on the sexual interest item than female patients upon admission to inpatient unit (Young et al., 2007). In our preclinical LHK rat model of mania, male rats showed a nonsignificant trend (*p* = .0516) toward increased sexual behavior compared with female rats (Abulseoud et al., 2015). Further studies are needed to investigate the phenomenology and neurobiological basis of sex differences in impulsive behaviors during manic episode.

Interestingly, manic females in our group were less appropriately dressed and groomed compared to males but they also had more insight into their illness. It is conceivable that self‐awareness drives both the motivation for better appearance and insight into mental illness. However, poor motivation and self‐care are also common during depression. Females in this study reported a nonsignificant trend (*p* = .09) toward more depressive symptoms during hospitalization which could explain, at least in part, the discrepancy between insight and overall appearance. In contrast to our results, a recent study (Guclu et al., 2011) reported male manic patients had better insight compared to female patients upon admission. However, that study used the Scale of Unawareness of Mental Disorders (SUMD; Amador, Strauss, Yale, & Gorman, 1991) developed to assess unawareness of illness in schizophrenia while we used question 11 of the YMRS. These two scales could be probing different aspects of disease awareness with SUMD more focused on insight into psychotic symptoms while YMRS question 11 is a general questionnaire about admitting or denying mental illness or behavioral changes. A meta‐analysis of eight studies looking at the change in insight ratings during hospitalization for acute mania found impaired insight upon admission and significant improvement at time of discharge (Ghaemi & Rosenquist, 2004). However, this meta‐analysis reported no data on sex difference. Insight and self‐awareness in general are higher cortical functions that are compromised in several psychiatric disorders (Sass & Parnas, 2003) and have a profound impact on psychosocial outcome (Ghaemi, Boiman, & Goodwin, 2000) but are challenging to study in preclinical models. Functional imaging studies on insight in patients with schizophrenia show different activation pattern in response to self‐ and/or other‐referential probes in medial orbitofrontal cortex, prefrontal cortex, and posterior cingulate (Blackwood et al., 2004; Holt et al., 2011; Shad et al., 2012). Only a few studies have looked at differences in activation pattern between manic patients and healthy controls (reviewed in Chen, Suckling, Lennox, Ooi, & Bullmore, 2011) without specifically addressing the neural network behind impaired insight during manic episode for sex difference.

Despite the differences in specific domains of mania between males and females, depressive and psychotic symptoms did not show sex difference and only a nonsignificant trend toward higher total MADRS scores was observed in females without any subject meeting DSM‐5 criteria of mixed episode (American Psychiatric Association, 2013). Future studies designed specifically to probe sex differences in the depressive pole of the illness are needed.

In addition, we found significant effect of sex on VPA dose where males received significantly higher doses compared to females. However, VPA plasma concentrations were not significantly different between the two groups, suggesting difference in body weight. Similarly, benzodiazepine dose equivalent showed slight overall effect for sex but did not survive post hoc multiple comparison test. In contrast, lithium dose, plasma concentration, and antipsychotic dose equivalent showed no sex differences. Taken together, it seems that both males and females received similar treatment and had comparable hospital LOS.

Remarkably, none of the patients in this study met DSM‐5 criteria for mixed state and despite that about half of the females in our cohort reported history of suicide attempts compared to only 10% of males, we observed only a nonsignificant trend (*p* = .09) toward higher MADRS scores in females throughout hospitalization. In general, mixed mania presentation is not common. The prevalence estimations for mixed episodes according to DSM‐IV were about 13% (Vieta & Morralla, 2010). In addition, bipolar women were not found to be different from men in the number of depressive episodes or the proportion of time spent in depression (reviewed in Diflorio & Jones, 2010). Furthermore, several studies found limited influence on male sex on suicide risk (reviewed in Diflorio & Jones, 2010) and one study reported that women were more likely to report history of suicidal attempts compared to men (Benedetti et al., 2007). Whether the sex difference in history of suicide attempts in this study is subject to recall bias or true vulnerability for bipolar women needs to be studied further.

To our surprise, the slight, but significant, elevation in heart rate and diastolic blood pressure was observed only in male patients. Bipolar type I patients are known to have higher mortality due to cardiovascular risk factors such as hypertension (Murray, Weiner, Prabhakar, & Fiedorowicz, 2009; Prieto et al., 2016). This increase in heart rate and diastolic blood pressure could stem from alterations in the autonomic nervous system (Nardelli, Valenza, Gentili, Lanata, & Scilingo, 2014) or activation of the hypothalamic–pituitary–adrenal axis (Watson, Gallagher, Ritchie, Ferrier, & Young, 2004). However, sex‐specific effects need to be further investigated in subsequent studies.

**Figure 2:**
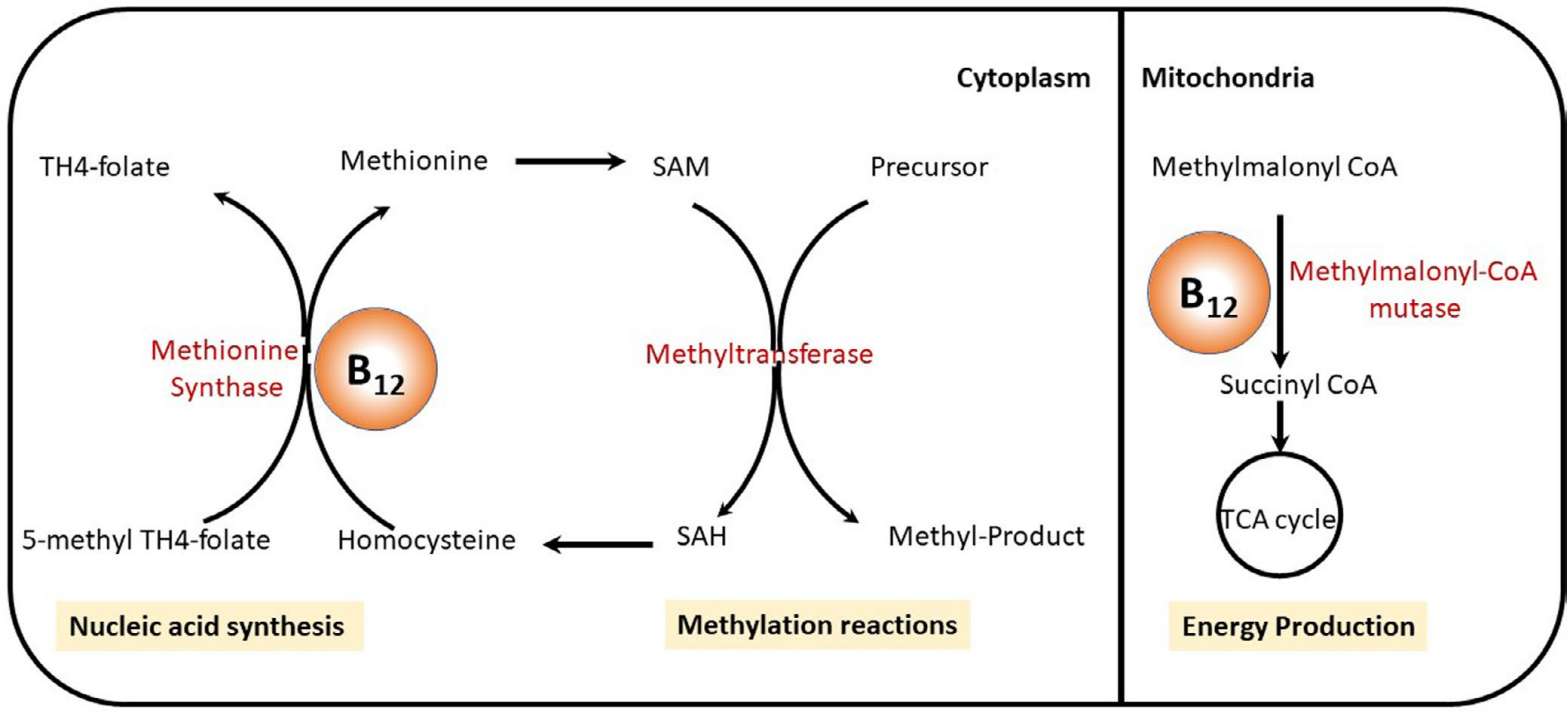
Vitamin B_12_ and folate have fundamental role in cellular metabolism. Vitamin B_12_ is a cofactor for cytoplasmic methionine synthase (MS) and mitochondrial methylmalonyl‐CoA (MMCo‐A) mutase enzymes. MS converts homocysteine into methionine (essential to sustain adequate synthesis of myelin, proteins, DNA, and neurotransmitters) and 5‐methyl tetrahydrofolate (TH4‐folate) into tetrahydrofolate (TH4) needed for nucleic acid synthesis (Calderon‐Ospina & Nava‐Mesa, 2020; Hathout & El‐Saden, 2011; Reynolds, 2006). Methionine is transformed into S‐Adenosyl Methionine (SAM) which is converted by methyltransferase into S‐Adenosyl Homocysteine (SAH) by the enzyme methyltransferase an important step in methylation reactions which is essential for genomic and nongenomic methylation. Inside the mitochondria, vitamin B_12_ functions as a cofactor for MMCo‐A mutase which converts methylmalonic acid to succinyl co‐enzyme A, which subsequently enters in the Krebs cycle for ATP production (Froese & Gravel, 2010; Gueant et al., 2013)

Interestingly, we found significant sex differences in several physiological measures (Table 3) most notably, low normal vitamin B_12_ concentration in males compared to females and low free T_3_ in both males and females compared to reference range, with females showing significantly lower concentrations compared to male patients. Vitamin B_12_ is an essential micronutrient that is obtained from animal dietary sources (Antony, 2003) and, along with folate, plays fundamental metabolic roles in keeping neuronal integrity and optimal functionality (see Figure 2 for more details; Calderon‐Ospina & Nava‐Mesa, 2020). Low plasma B_12_ concentration is common in the general population (Zucker, Livingston, Nakra, & Clayton, 1981), and for unknown reasons, males have lower levels of B_12_ compared to females (Fernandes‐Costa, Tonder, & Metz, 1985; Low‐Beer, McCarthy, Austad, Brzechwa‐Ajdukiewicz, & Read, 1968; Metz, Hart, & Harpending, 1971). Decreased plasma B_12_ levels present clinically with megaloblastic anemia, subacute combined degeneration, and other neuropsychiatric manifestations specifically mood disorder (Mitchell, Conus, & Kaput, 2014; Shorvon, Carney, Chanarin, & Reynolds, 1980). Furthermore, several cases of severe bipolar mania, hypomania, and mixed states have been reported in patients with B_12_ deficiency with symptomatic recovery after vitamin supplementation (Durand, Mary, Brazo, & Dollfus, 2003; Goggans, 1984; Gomez‐Bernal & Bernal‐Perez, 2007; Tufan, Bilici, Usta, & Erdoğan, 2012).

Since we measured B_12_ levels only upon admission, it is not clear whether the low normal concentration in our cohort is a transient state during the manic episode or a stable trait for bipolar patients. It could be that the vulnerability to low vitamin B_12_ concentrations increases in the presence of genetic polymorphism of metabolizing enzymes (trait) or due to exposure to medications that are known to alter enzyme activity (state). Nitrous oxide exposure, for example, suppresses MS activity and causes rapid reduction in vitamin B_12_ concentration (reviewed in Hathout & El‐Saden, 2011; Reynolds, 2006). Similarly, VPA causes reduction in MS activity (Alonso‐Aperte, Ubeda, Achon, Perez‐Miguelsanz, & Varela‐Moreiras, 1999) and methionine concentration (Ubeda, Alonso‐Aperte, & Varela‐Moreiras, 2002) but without reduction in vitamin B_12_ concentration. Dopamine, on the other hand, activates MS (Waly et al., 2004) and one could speculate that dopamine receptor antagonists (i.e., antipsychotics) could have an opposite effect and hence suppress MS activity. However, this remains as mere speculation that requires further studies to investigate the relationship between mood state, antimanic medications and plasma vitamin B_12_ concentration and whether low vitamin B_12_ associated alteration in DNA methylation, gene expression and other epigenetic mechanisms impacts symptom presentation.

**Figure 3:**
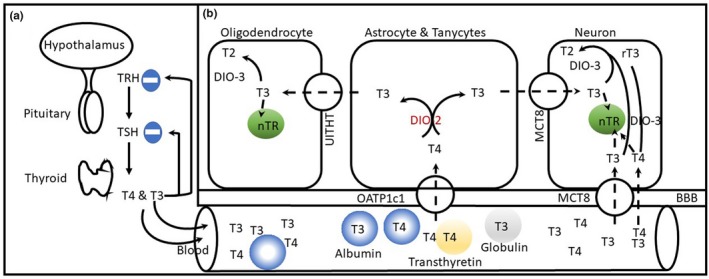
Brain thyroid hormone. (a) Hypothalamic–pituitary–thyroid (HPT) axis; thyrotropin‐releasing hormone (TRH) is released from the hypothalamus to stimulate anterior pituitary to secrete thyroid stimulating hormone (TSH) which in turn stimulates thyroid gland to produce mostly tetraiodothyronine (T4) and to less extent triiodothyronine (T3). (b) Transportation and brain action of plasma T3 and T4: Both T3 and T4 are present in free and protein‐bound forms. Free T4 is transported through the blood–brain barrier (BBB) by the organic anion‐transporting peptide 1c1 (OATP1c1) to astrocytes and tanycytes where it is converted into T3 by deiodinase type 2 (DIO‐2) enzyme. Astrocyte T3 is transported to neurons through monocarboxylate transporter (MCT)8. Free T3 and to less extent T4 can also be transported directly into neurons through gap junctions and MCT8. Another unknown thyroid hormone transporter (UTHT) transporters astrocytic T3 to oligodendrocytes to activate myelination genes. Within neurons, T3 (and to possibly T4) bind to nuclear thyroid hormone receptors (nTR) to influence gene expression critical for cell growth and differentiation and synaptic plasticity. Neuronal T4 and T3 are metabolized by deiodinase type 3 (DIO‐3) enzyme into inactive reverse T3 (rT3) and T2, respectively (Cheng, Leonard, & Davis, 2010; Lee & Petratos, 2016; Schroeder & Privalsky, 2014)

In addition to the low normal B_12_ concentration, we observed low free T3 concentrations and within reference range TSH and free T4 levels. Our results are in agreement with Kirkegaard, Bjørum, Cohn, and Lauridsen (1978) who reported decrease in serum T3 level and free T3 index during mania and with Cassidy, Ahearn, and Carroll (2002) report of no difference in TSH or FT4 concentrations between manic and mixed episodes and normal reference values. Other studies found increased free T4 index in mixed mania (Joffe, Young, Cooke, & Robb, 1994) and blunted TSH response to TRH challenge in manic patients (Extein et al., 1980). Collectively, these results suggest that acute mania is associated with altered peripheral thyroid indices. However, we cannot role an effect for antipsychotic medications on the low FT3 levels we observed (Bunevicius, Steibliene, & Prange, 2014). Regardless to the basis for the change in FT3, and given the significant role of optimal thyroid economy on brain function (see Figure 3 for details), further studies are needed to investigate thyroid dynamic changes during acute manic episode and its relationship with sex‐specific variability in mania phenotype.

The results of this study should be viewed in the light of its limitations and strength. The study was conducted in Turkey where alcohol and drug comorbidities are less reported compared to western countries. The number of female participants who reported history of alcohol use was not large enough to use it as a covariate in the analysis. However, future studies are needed to test the validity of our results in other non‐HMO western European countries. Here, it is relevant to note that we aimed to design this study in a non‐HMO facility where the hospital LOS is typically longer (Blader, 2011). Manic patients in our cohort were hospitalized for an average of about 3 weeks which is consistent with the average LOS in European countries and Turkey (Karamustafalioglu et al., 2014).

Interestingly, patients in our cohort received relatively high doses of antipsychotic medications (about 900 mg/day of Chlorpromazine equivalent) in conjunction with lithium and VPA. This complex pharmacotherapy strategy is not unusual when treating severe mania during acute hospitalization (Zarate & Quiroz, 2003). Zarate and Quiroz reviewed controlled trials looking at treatment modalities for acute mania and reported that the most useful treatment is the combination of antipsychotics with mood stabilizers: lithium and VPA (Zarate & Quiroz, 2003). Typically, the medication regimen is simplified during post discharge outpatient follow‐up visits.

The effort to enroll 57 acutely manic patients in this pilot naturalistic study and track the progression of their symptoms on daily basis is remarkable compared to the few other studies reporting sex differences, which were mostly retrospective and cross‐sectional in nature (Hendrick et al., 2000; Kumar & Ram, 2001; Viguera et al., 2000; Young et al., 2007). However, future studies should achieve larger cohort sizes to permit for examining the relationship between individual treatments and outcome of specific symptoms domains.

In conclusion, we found significant sex differences in sexual interest, aggressive and disruptive behavior, appearance and grooming, and insight item scores on the YMRS in acute manic patients. Total YMRS scores did not differ between males and females. Further studies are needed to address the neurobiological basis for sex difference in mania phenotype.

## CONFLICT OF INTEREST

The authors have no conflict of interest to declare.

## AUTHORS' CONTRIBUTION

OAA conceived the study. OAA, GS, OS, and UC designed the study. GS and OS collected the data. OAA and BS analyzed the data and wrote the first draft. All authors contributed to data interpretation, manuscript writing, and revisions.

## Data Availability

Data files are available upon request.
